# Assessing Sarcopenia, Presarcopenia, and Malnutrition in Axial Spondyloarthritis: Insights from a Spanish Cohort

**DOI:** 10.3390/nu17061019

**Published:** 2025-03-14

**Authors:** Laura Berbel-Arcobé, Diego Benavent, Lidia Valencia-Muntalà, Carmen Gómez-Vaquero, Xavier Juanola, Joan M. Nolla

**Affiliations:** Rheumatology Department, Hospital Universitari de Bellvitge-IDIBELL, Carrer de la Feixa Llarga S/N, L’Hospitalet de Llobregat, 08907 Barcelona, Spain

**Keywords:** sarcopenia, EWGSOP-2, spondyloarthritis, GLIM, malnutrition, comorbidity

## Abstract

Background/Objectives: Sarcopenia, defined by a loss of muscle mass, strength, and function, is a potential comorbidity in axial spondyloarthritis (axSpA). Its prevalence, along with malnutrition, remains unclear. Methods: This cross-sectional study assessed sarcopenia (using the European Working Group on Sarcopenia in Older People (EWGSOP-2) criteria), presarcopenia, and malnutrition (using the Global Leadership Initiative on Malnutrition (GLIM) criteria) in a Spanish axSpA cohort. We included 94 patients aged ≥ 50 years. Sarcopenia was evaluated using the SARC-F questionnaire and by measuring muscle strength, mass, and performance. Presarcopenia was defined as low muscle mass alone. Malnutrition was assessed using body mass index (BMI) and fat-free mass index (FFMI). Results: The prevalence of sarcopenia, presarcopenia, and malnutrition was 3.2%, 23.4%, and 10.6%, respectively. Sarcopenia correlated with worse functionality and quality of life (Bath Ankylosing Spondylitis Functional Index (BASFI) 7.6 ± 1.2 vs. 3.6 ± 2.5, *p* = 0.02; ASAS Health Index (ASAS-HI) 11 ± 2 vs. 5.6 ± 3.7, *p* = 0.03). Presarcopenia was linked to a lower BMI (24.7 ± 4.1 vs. 29.1 ± 4.2, *p* < 0.01), FFMI (16.1 ± 2 vs. 19.6 ± 2.6, *p* < 0.01), and reduced biologic treatment use (31.8% vs. 61.1%, *p* = 0.03). Malnourished patients had lower muscle mass (5.14 ± 0.73 vs. 6.23 ± 0.96, *p* < 0.01). SARC-F showed 100% sensitivity and 75.8% specificity for sarcopenia detection. Conclusions: Despite low sarcopenia prevalence, presarcopenia and malnutrition are frequent, highlighting the need for early detection in axSpA.

## 1. Introduction

Axial spondyloarthritis (axSpA) is a chronic inflammatory rheumatic disease primarily affecting the vertebral spine and sacroiliac joints. Structural damage, alongside other clinical manifestations and comorbidities, contributes to functional impairment and a reduced health-related quality of life [1,2]. 

Sarcopenia is an emerging comorbidity in patients with chronic rheumatic diseases, contributing to functional decline, disability, and adverse health outcomes [3]. Its development is multifactorial, driven by systemic inflammation, physical inactivity, chronic corticosteroid use, and metabolic alterations inherent to conditions such as axSpA [4], rheumatoid arthritis [5], and systemic lupus erythematosus [6]. Early recognition and targeted interventions are essential to mitigate its impact and preserve musculoskeletal function in this vulnerable population.

Currently, although there is no universally accepted operational definition of sarcopenia, the most widely recognized criteria are the 2019 guidelines proposed by the European Working Group on Sarcopenia in Older People (EWGSOP), referred to as the EWGSOP-2 criteria [7]. This diagnostic framework follows a sequential approach, beginning with case-finding using the SARC-F questionnaire [8] to identify individuals at risk. In these individuals, sarcopenia is assessed stepwise by evaluating muscle strength, muscle quantity, and physical performance, allowing classification into probable, confirmed, and severe sarcopenia. This structured process ensures accurate identification and stratification to guide clinical management.

On the other hand, presarcopenia, a term often used to describe the early stages of muscle health decline, is characterized by a reduction in muscle mass without accompanying deficits in muscle strength or physical performance [9]. While the EWGSOP-2 criteria do not specifically classify presarcopenia as a distinct diagnostic category, its evaluation may provide meaningful perspectives into the initial stages of musculoskeletal deterioration. By assessing presarcopenia, we can capture individuals at an earlier stage in the sarcopenic continuum, potentially identifying those at higher risk for progression to sarcopenia and its associated adverse outcomes. This approach complements the EWGSOP-2 framework, offering a broader perspective on muscle health and informing preventive and therapeutic strategies to mitigate long-term disability.

Malnutrition related to disease is another critical, yet often overlooked, challenge in healthcare, especially among patients with chronic rheumatic diseases who exhibit increased susceptibility [10,11]. Until recently, malnutrition lacked standardized diagnostic criteria, contributing to inconsistencies and under recognition in clinical practice. In response to this issue, the Global Leadership Initiative on Malnutrition (GLIM) introduced consensus diagnostic guidelines in 2018, establishing a standardized framework incorporating phenotypic criteria (weight loss, low body mass index, reduced muscle mass) and etiological criteria (reduced intake/absorption, inflammatory disease burden) [12]. The GLIM criteria provide a robust clinical tool enabling accurate identification and classification of malnutrition, facilitating timely interventions to improve patient outcomes. While sarcopenia and malnutrition are a potential concern in patients with axSpA [4], its true prevalence remains unclear. 

The aim of the present study is to analyze the prevalence of sarcopenia, presarcopenia, and malnutrition in a cohort of Spanish patients with axSpA. Additionally, this research seeks to evaluate the effectiveness of the SARC-F questionnaire in detecting sarcopenia within this demographic. Ultimately, our objective is to explore the clinical relevance of these conditions and consider the potential benefits of incorporating their systematic evaluation into routine clinical practice.

## 2. Materials and Methods

### 2.1. Study Population

This observational, cross-sectional study enrolled patients who met the Assessment of Spondyloarthritis International Society (ASAS) criteria for axSpA. Given that sarcopenia is commonly associated with age [13], patients over 50 years old were included. Recruitment took place from November 2022 to March 2024 during routine visits to the Department of Rheumatology of the Bellvitge University Hospital, a tertiary university hospital. Patients with severe disability (wheelchair-bound) or an occiput-to-wall distance greater than 10 cm were excluded, as these conditions would hinder the feasibility of conducting sarcopenia assessment methods as well as whole-body densitometry (DXA). Patients with conditions likely to significantly influence their clinical status, such as neoplasms, cardiac or respiratory insufficiency, or chronic liver or kidney disease, were also excluded. All participants provided written informed consent, and the study received approval from the local ethics committee (reference: PR329/22).

### 2.2. Study Variables

#### 2.2.1. Sociodemographic and Anthropometric Data

The following data were collected:

- Gender;
- Age;
- Body mass index (BMI): BMI was calculated as weight in kilograms and was categorized as underweight (<18.5 kg/m^2^), normal range (18.5–24.9 kg/m^2^), overweight (25–29.9 kg/m^2^), and obese (≥30 kg/m^2^);
- Tobacco use: Patients were classified into three groups based on tobacco use history: never smokers, current smokers, and former smokers. For the purposes of this study, we defined “never smokers” as including both current and former smokers;
- Physical activity: Physical activity was categorized according to participants’ self-reported engagement in regular exercise;
- History of fragility fracture: This was defined as a fracture resulting from minimal trauma, such as a fall from standing height or less, indicating underlying bone fragility. This variable captures any self-reported history of such fractures.

#### 2.2.2. axSpA Assessment

- axSpA history: (a) disease duration in months; (b) axSpA subtype, classified as radiographic or non-radiographic, (c) HLA-B27 status; (d) articular manifestations (peripheral arthritis, enthesitis, dactylitis) and extra-articular manifestations (uveitis, psoriasis, onychopathy and chronic inflammatory bowel disease); and (e) current treatment regimen: nonsteroidal anti-inflammatory drugs (NSAIDs), history of glucocorticoids use, conventional synthetic disease-modifying anti-rheumatic drugs (csDMARDs), and biologic disease-modifying anti-rheumatic drugs (bDMARDs).
- Analytical evaluation: hemoglobin and C-reactive protein (CRP) values were considered; the values corresponding to the last analytical study carried out were considered.
- Activity analysis: this was assessed using the following two established indices:
  ○ Bath Ankylosing Spondylitis Disease Activity Index (BASDAI) [14]: The BASDAI is a self-reported measure that evaluates fatigue, spinal pain, joint pain/swelling, enthesitis, and morning stiffness in patients with axSpA. Scores range from 0 to 10, with higher scores indicating greater disease activity.
  ○ Ankylosing Spondylitis Disease Activity Score with C-reactive Protein (ASDAS-CRP) [15]: The ASDAS-CRP combines patient-reported symptoms with CRP levels, an objective inflammatory marker, to provide a composite score of disease activity. ASDAS-CRP values are categorized as follows: (a) <1.3: inactive disease, (b) 1.3–2.1: low disease activity; (c) 2.1–3.5: high disease activity; (d) >3.5: very high disease activity.
- Evaluation of disability: This was assessed using the Bath Ankylosing Spondylitis Functional Index (BASFI) [16]. The BASFI is a validated, self-administered tool designed to measure the impact of axSpA on a patient’s physical function in daily life. It consists of 10 items that focus on specific functional activities, such as bending, dressing, standing, and reaching. Each activity is rated by the patient on a 0 to 10 scale, where 0 indicates no difficulty and 10 signifies severe limitation.
- Evaluation of the health-related quality of life: for this, we used two questionnaires, namely a disease-specific measure, the ASAS Health Index (ASAS-HI), and a general measure, the Short Form Health Survey (SF-12), as follows:
  ○ ASAS-HI: The ASAS-HI [17] is a targeted instrument specifically designed to assess the impact of axSpA on various aspects of health and daily living. It includes 17 dichotomous items (yes/no) addressing physical, emotional, and social domains, giving a comprehensive view of how axSpA affects patients’ lives. The total score ranges from 0 to 17, with higher scores indicating greater health impact and disability related to axSpA.
  ○ SF-12: The SF-12 [18] is a concise, 12-item questionnaire designed to evaluate health-related quality of life from the patient’s perspective. It captures both physical and mental health dimensions and provides two composite scores, one for physical health and one for mental health. This shortened version of the SF-36 survey reduces the respondent burden while maintaining essential insights into overall health status. Each score ranges from 0 to 100, with higher scores reflecting better health-related quality of life.

#### 2.2.3. Sarcopenia Assessment

- Muscle strength: This was measured using a calibrated handheld dynamometer (Kern hand grip digital dynamometer 80K1), with two trials performed for each hand. The highest recorded value from the stronger hand was used. A cutoff of <27 was applied to identify reduced grip strength in men and <16 kg in women, as defined by the EWGSOP-2 criteria.
- Gait speed: Physical performance was assessed using the 6 m gait speed test. Participants walked a straight 6 m path, and the time was recorded using a stopwatch. Gait speed was calculated in meters per second (m/s), with <0.8 m/s considered indicative of limited physical performance, based on the EWGSOP-2 standard.
- Muscle mass: Skeletal muscle mass was measured by calculating the Skeletal Mass Index (SMI) using the following formula: appendicular skeletal muscle mass/height^2^. Measurements were taken using a Hologic Horizon W densitometer (Hologic Inc., Bedford, MA), recording lean and fat mass in the arms, trunk, and legs. The patient was positioned supine on the examination table, arms extended at their sides with hands facing the legs but not touching, and thumbs pointing upward. The cutoff for low muscle mass, based on EWGSOP-2 criteria, was set at <7 kg/m^2^ in men and <5.5 kg/m^2^ in women.
- SARC-F: The SARC-F questionnaire [8] was used as an initial screening tool, following EWGSOP-2 recommendations. This tool includes five components: strength, need for assistance walking, rising from a chair, climbing stairs, and history of falls, with a score ranging from 0 to 10. A score of ≥4 suggests the presence of sarcopenia and the need for further evaluation.
- Definition of sarcopenia: Confirmed sarcopenia was diagnosed when low muscle strength was accompanied by low muscle mass in patients with a SARC-F score ≥ 4. Severe sarcopenia was identified in cases with sarcopenia that additionally exhibited poor physical performance.
- Definition of presarcopenia: presarcopenia was defined as the presence of low muscle mass (SMI < 7 kg/m^2^ in men and <5.5 kg/m^2^ in women) without impairments in muscle strength or physical performance.

#### 2.2.4. Nutritional Assessment

- Malnutrition was defined according to GLIM criteria [12]. Reduced muscle mass was identified by a fat-free mass index (FFMI, kg/m^2^) of below 16.7 in males and 14.6 in females. A low BMI was defined as <20 kg/m^2^, or <22 kg/m^2^ for individuals aged 70 years or older.

### 2.3. Statistical Analysis

We calculated the necessary sample size based on a 5% expected prevalence of sarcopenia in axSpA [19], using the following formula: *n* = Z^2^ × p × (1 − p)/E^2^. Here, Z is the Z-score for a 95% confidence level (1.96), *p* is the prevalence rate (0.05), and E is the margin of error (5%). This calculation yielded a required sample size of 73 participants.

In the baseline descriptive study, categorical variables were presented as the number and percentage of subjects in each category. Continuous variables were described as mean and standard deviation or median and interquartile range, depending on the distribution.

Patients were categorized into two distinct groups based on the presence or absence of sarcopenia, presarcopenia, and malnutrition. Group differences were analyzed using the chi-squared test or Fisher’s test for categorical variables. The normality of quantitative variables was verified using the Kolmogorov–Smirnov test. For quantitative variables that met the normality criteria, the Student’s *t*-test was used, while non-parametric tests (Mann–Whitney U) were applied for variables that did not meet the normality criteria. 

We also conducted a comprehensive evaluation of the effectiveness and precision of SARC-F. Its sensitivity, specificity, positive predictive value (PPV), negative predictive value (NPV), and diagnostic accuracy were assessed. Sensitivity measures the proportion of true positive cases correctly identified by the test among all actual positive cases. Specificity assesses the test’s ability to correctly identify negative cases among those without the studied condition. PPV represents the probability that a positive test result is true. NPV indicates the probability that a negative test result is true. Diagnostic accuracy reflects the proportion of true results, both positive and negative, in relation to the total results obtained.

All analyses were conducted using R (version 4.2.2), and the significance level was set at *p* < 0.05.

## 3. Results

The study population comprised 94 patients, with 73.4% (*n* = 69) being men. The mean age was 64.4 ± 9.1 years, and the mean BMI was 28.1 ± 4.6 kg/m^2^. A history of smoking was reported by 53.2% (*n* = 50), and 23.4% (*n* = 22) engaged in regular physical exercise. Fragility fractures were recorded in 6.4% (*n* = 6) of the patients. 

The mean disease duration was 26.1 ± 13.8 months. Among the cohort, 78.7% (*n* = 74) were diagnosed with radiographic axSpA, and 78.7% (*n* = 74) were HLA-B27 positive. During their clinical course, uveitis occurred in 29.8% (*n* = 28), psoriasis in 11.7% (*n* = 11), onychopathy in 2.1% (*n* = 2), and inflammatory bowel disease in 12.8% (*n* = 12). A total of 44.7% (*n* = 42) patients presented with peripheral arthritis, 25.5% (*n* = 24) suffered from enthesitis, and 5.3% (*n* = 5) had dactylitis. 

Regarding treatment, 70.2% (*n* = 66) were receiving NSAIDs, 6.4% (*n* = 6) had a history of glucocorticoids use, 4.3% (*n* = 4) were treated with csDMARDs, and 54.3% (*n* = 51) were treated with bDMARDs. The mean hemoglobin level was 146 ± 16.7 g/L, and the CRP level was 3.5 ± 2.2 mg/L. Disease activity scores showed a mean BASDAI of 3.5 ± 2.2 and an ASDAS-CRP of 2.1 ± 0.9. Functional status measured with BASFI had a mean score of 3.8 ± 2.6, while quality of life scores averaged 5.8 ± 3.8 for ASAS-HI, 40.4 ± 10.8 for the physical component of SF-12 questionnaire, and 49.8 ± 11.3 for the mental component. The mean SARC-F questionnaire score was 2.3 ± 2.1, and 26.6% (*n* = 25) of patients scored ≥4. 

Confirmed sarcopenia was identified in 3.2% (*n* = 3) of the study population; none of these patients had inflammatory bowel disease. Severe sarcopenia was not observed in any participant. Patients with sarcopenia were younger compared to those without sarcopenia (51.7 ± 1.2 vs. 64.8 ± 8.9, *p* < 0.01) and had lower hemoglobin levels (127.3 ± 5.5 vs. 146.6 ± 16.6, *p* = 0.01), poorer functionality and quality of life (BASFI 7.6 ± 1.2 vs. 3.6 ± 2.5, *p* = 0.02; ASAS-HI 11 ± 2 vs. 5.6 ± 3.7, *p* = 0.03), and worse scores in both the physical component of the SF-12 questionnaire and the SARC-F questionnaire (physical health according to SF-12 results 23.5 ± 4.5 vs. 40.9 ± 10.5, *p* = 0.01; SARC-F 6 ± 1 vs. 2.2 ± 2, *p* = 0.01). The complete characteristics of the patients with axSpA and the differences between those without and with sarcopenia are shown in Table 1.

Presarcopenia was found in 23.4% (*n* = 22) of patients. Table 2 compares the clinical and demographic characteristics of patients without and with presarcopenia. In the comparative analysis, patients with presarcopenia had a lower BMI (24.7 ± 4.1 vs. 29.1 ± 4.2, *p* < 0.01) and FFMI (16.1 ± 2 vs. 19.6 ± 2.6, *p* < 0.01). Moreover, fewer patients were treated with bDMARDs (31.8% vs. 61.1%, *p* = 0.03). No statistically significant differences were observed in other variables.

The prevalence of malnutrition was 10.6% (10/94). Nine patients (two men and seven women) met the FFMI-related criteria, whereas only one female patient met the BMI-related criteria; this patient also fulfilled the FFMI-related criteria as the tenth patient to do so. Patients classified as malnourished exhibited a significantly lower muscle mass (SMI) than those without malnutrition (5.14 ± 0.73 vs. 6.23 ± 0.96; *p* < 0.01).

The diagnostic performance of the SARC-F questionnaire for sarcopenia detection (based on EWGSOP2 criteria) is detailed in Table 3.

## 4. Discussion

We aimed to determine the prevalence of sarcopenia, presarcopenia, and malnutrition in a cohort of Spanish patients with axSpA and to evaluate the effectiveness of the SARC-F questionnaire in detecting sarcopenia within this demographic. Our findings showed that presarcopenia was notably more frequent than sarcopenia or malnutrition, although each showed important clinical correlations. Sarcopenia, observed at a prevalence of 3.2%, was associated with poorer functionality, reduced hemoglobin levels, and a lower quality of life. Conversely, presarcopenia was present in nearly one-quarter of the cohort and was linked to a lower BMI, a reduced fat-free mass index, and the less frequent use of biologic therapy. Furthermore, 10.6% of patients were classified as malnourished, all of whom exhibited a significantly lower muscle mass. Our findings suggest that presarcopenia appears to be associated with malnutrition, as patients with presarcopenia had a significantly lower FFMI, and those meeting the GLIM criteria for malnutrition exhibited a lower muscle mass compared to those without malnutrition. Moreover, the SARC-F questionnaire demonstrated high sensitivity and specificity in detecting sarcopenia in our cohort, and appears valuable for quickly identifying patients who may require further assessment.

Our findings reveal a relatively low prevalence of confirmed sarcopenia, contrasting with the higher rates observed in other chronic inflammatory diseases [20,21] and axSpA.

A systematic review by Ceolin et al. [22] reported sarcopenia prevalence rates in ankylosing spondylitis (AS) ranging from 0% to 34%, depending on the diagnostic criteria applied. The authors noted that muscle strength impairments were more commonly observed than reductions in muscle mass, suggesting that strength deficits may represent a more sensitive marker of sarcopenia in this population. Furthermore, the review identified an association between higher AS disease activity and an increased prevalence of sarcopenia, highlighting the role of chronic inflammation in its pathogenesis. Similarly, a meta-analysis by Hu et al. [23] estimated an overall prevalence of sarcopenia in spondyloarthritis patients of 25% (95% CI: 12.7–35.2%), with severe sarcopenia observed in 8.7% of cases. Aguiar et al. [24] reported a prevalence of sarcopenia of 62% in SpA patients, including those with peripheral involvement, using the Lee equation to assess muscle mass relative to a control group. El Maghraoui et al. [25] found presarcopenia and sarcopenia rates of 50.4% and 34.3%, respectively, in Moroccan AS patients, employing DXA and physical performance tests per EWGSOP criteria. In Asian populations, Kanjanavaikoon et al. [26] documented a 22.1% prevalence of sarcopenia in axSpA using the Asian Working Group for Sarcopenia criteria, which included DXA and the SARC-F questionnaire, an approach similar to ours, but yielding slightly higher rates. In contrast, Neto et al. [27], in the MyoSpA study, identified no sarcopenia in young axSpA patients according to EWGSOP2, despite observing notable muscle dysfunction. Our results align closely with those reported by Merle et al. [19] in the SASPAR study, which evaluated sarcopenia in 103 French patients with SpA (51% women) using a methodology closely aligned with the EWGSOP-2 strategy. Their cohort had a mean age of 47.1 ± 13.7 years and included 51% axSpA and 49% peripheral SpA. The study reported a prevalence of 5% for confirmed sarcopenia and 21% for probable sarcopenia, defined as low grip strength. All sarcopenic patients exhibited low gait speed, indicative of severe sarcopenia. While the authors describe their approach as based on the EWGSOP-2 framework, the absence of the initial SARC-F screening step slightly differentiates their methodology from full compliance with EWGSOP-2 criteria. 

In terms of presarcopenia, our results are consistent with those described in various studies. The meta-analysis by Hu et al. [23] reported a pooled prevalence of presarcopenia in SpA patients at 21% and identified associations with chronic inflammation. Similarly, Ceolin et al. [22], in their systematic review, reported presarcopenia prevalence rates ranging from 20% to 30% in SpA, noting strong links to elevated disease activity and reduced physical performance, even in the absence of sarcopenia. Both studies emphasize the importance of recognizing presarcopenia as a possible precursor to confirmed sarcopenia, representing an intermediate stage in the continuum of muscle health deterioration. 

Regarding the nutritional status of patients with axSpA, existing data are limited, and the true extent of the problem is not fully understood. Furthermore, malnutrition assessment remains infrequent in routine clinical practice. Recently, a study conducted in Sweden by Hulander et al. [28], including 155 patients with radiographic axSpA, demonstrated an impaired dietary intake in these patients. Notably, a lower intake of several nutrients with potential anti-inflammatory properties—such as fiber, marine omega-3 fatty acids, vitamin D, and selenium—was observed. On the other hand, a large multicenter study in China by Li et al. [29], which included 4146 patients, found that 11.99% had a low BMI, which was significantly associated with elevated CRP levels and higher BASFI scores. All these findings, together with our results, reinforce the need to incorporate nutritional assessment into the clinical follow-up of axSpA patients. The early detection and correction of deficiencies can support the maintenance of muscle mass and help mitigate inflammation. Optimizing dietary intake—especially by ensuring sufficient protein within a balanced Mediterranean-style diet—may be beneficial, and tailored nutritional supplementation should be considered when necessary. Additionally, a multidisciplinary approach involving nutritionists and physiotherapists is crucial for developing personalized dietary and exercise plans, as well as consistently monitoring patient progress.

These findings emphasize significant variability across studies regarding the prevalence of sarcopenia, largely due to differences in study populations and diagnostic methodologies, reinforcing the need for standardized frameworks to improve comparability and reliability in sarcopenia research. Our cohort exhibited primarily axial involvement, relatively well-controlled disease activity, and a high proportion of patients on biologic therapy, which may mitigate muscle loss. Moreover, variations in age, comorbidities, and physical activity could further account for these discrepancies. In contrast, some studies used alternative measurement techniques, while we evaluated the prevalence of sarcopenia using the EWGSOP-2 criteria, the current gold standard for defining the condition. To the best of our knowledge, this is the first application of these criteria in the context of axSpA, providing a systematic and reliable framework for assessing muscle health in this specific population. By systematically applying the EWGSOP-2 criteria, our study provides a more accurate and standardized estimation, clarifying its actual impact in this population and avoiding potential overestimation or misclassification.

Our study has several limitations. First, its cross-sectional design prevents us from establishing causal relationships or evaluating long-term outcomes. Therefore, longitudinal studies will be necessary. Second, single-center recruitment may reduce generalizability, although it reflects the reality of cohorts in university hospitals previously reported [30]. Third, the exclusive inclusion of patients over 50 years may overlook younger axSpA patients also at risk. Fourth, the absence of a control group complicates distinguishing the specific impact of axSpA on muscle health and malnutrition. Finally, the small number of patients with sarcopenia limits statistical power, as well as the ability to detect subtle associations; nevertheless, the use of rigorous EWGSOP-2 criteria ensures reliable diagnoses and offers valuable preliminary insights into sarcopenia in axSpA, laying the groundwork for future research with larger cohorts.

Nonetheless, the study has significant strengths. Foremost, it is the first to systematically apply the EWGSOP-2 and GLIM criteria exclusively to axSpA patients. This approach provides a robust framework for identifying and classifying sarcopenia, presarcopenia, and malnutrition, enhancing reliability and reproducibility for future research. 

## 5. Conclusions

Our study reveals a notable prevalence of presarcopenia among axSpA patients, while sarcopenia itself is less frequent. Sarcopenia was associated with reduced functional capacity and lower health-related quality of life. Although no statistically significant differences were found between patients with and without presarcopenia in terms of disease activity, functionality, or quality of life, its prevalence highlights its potential clinical relevance as an early marker of muscle health deterioration. Additionally, we found a substantial prevalence of malnutrition among these patients. 

In conclusion, integrating muscle health and nutritional assessments into axSpA management strategies represents a practical and impactful approach to improving long-term patient outcomes.

## Figures and Tables

**Table 1:** Characteristics of the patients with axSpA and differences between the ones without and with sarcopenia.

	All Patients (*n* = 94)	Without Sarcopenia (*n* = 91)	With Sarcopenia (*n* = 3)	*p*
Sociodemographic and anthropometric data				
Male	69 (73.4%)	68 (74.7%)	1 (33.3%)	ns
Mean age (years)	64.4 ± 9.1	64.8 ± 8.9	51.7 ± 1.2	<0.01
BMI (kg/m^2^)	28.1 ± 4.6	28.2 ± 4.6	25 ± 2	ns
Normal	16 (17%)	15 (16.5%)	1 (33%)	
Overweight	48 (51.1%)	46 (50.5%)	2 (66%)	
Obese	30 (31.9%)	30 (32.9%)	0 (0%)	
Never smokers	50 (53.2%)	49 (53.8%)	1 (33.3%)	ns
Regular exercise	22 (23.4%)	22 (24.2%)	0	ns
Fragility fracture	6 (6.4%)	6 (6.6%)	0	ns
axSpA and sarcopenia assessment				
Disease duration (months)	26.1 ± 13.8	25.8 ± 13.8	33.6 ± 12.5	ns
r-axSpA	74 (78.7%)	71 (78%)	3 (100%)	ns
HLA-B27-positive	74 (78.7%)	71 (78%)	3 (100%)	ns
Articular symptoms				ns
Peripheral arthritis	42 (44.7%)	41 (45.1%)	1 (33.3%)	
Enthesitis	24 (25.5%)	22 (24.2%)	2 (66.7%)	
Dactylitis	5 (5.3%)	5 (5.5%)	0	
Extra-articular symptoms				ns
Uveitis	28 (29.8%)	27 (29.7%)	1 (33.3%)	
Psoriasis	11 (11.7%)	11 (12.1%)	0	
Onychopathy	2 (2.1%)	2 (2.2%)	0	
IBD	12 (12.8%)	12 (13.2%)	0	
Current treatment				ns
NSAID	66 (70.2%)	63 (69.2%)	3 (100%)	
Glucocorticoids	6 (6.4%)	6 (6.6%)	0	
csDMARDs	4 (4.3%)	4 (4.4%)	0	
bDMARDs	51 (54.3%)	50 (54.9%)	1 (33.3%)	
Hemoglobin (g/L)	146 ± 16.7	146.6 ± 16.6	127.3 ± 5.5	0.01
CRP (mg/L)	3.5 ± 7.1	3.5 ± 7.2	3.8 ± 4	ns
FFMI	18.8 ± 2.9	18.9 ± 2.9	17.2 ± 1.5	ns
BASDAI	3.5 ± 2.2	3.4 ± 2.2	4.9 ± 1.8	ns
ASDAS-CRP	2.1 ± 0.9	2.1 ± 0.9	2.7 ± 0.8	ns
Inactive	18 (19.1%)	18 (19.8%)	0	
LDA	34 (36.2%)	34 (37.4%)	0	
HDA	34 (36.2%)	32 (35.2%)	2 (66%)	
VHDA	8 (8.5%)	7 (7.7%)	1 (33%)	
BASFI	3.8 ± 2.6	3.6 ± 2.5	7.6 ± 1.2	0.02
ASAS-HI	5.8 ± 3.8	5.6 ± 3.7	11 ± 2	0.03
SF-12				
Mental health	49.8 ± 11.3	49.8 ± 11.4	52.7 ± 2.1	ns
Physical health	40.4 ± 10.8	40.9 ± 10.5	23.5 ± 4.5	0.01
SARC-F	2.3 ± 2.1	2.2 ± 2	6 ± 1	0.01

Results are expressed as *n* (%) or mean ± SD. BMI: body mass index; axSpA: axial spondyloarthritis; r-axSpA: radiographic axial spondyloarthritis; IBD: inflammatory bowel disease; NSAID: nonsteroidal anti-inflammatory drugs; csDMARDs: conventional synthetic disease-modifying anti-rheumatic drugs; bDMARDs: biologic disease-modifying anti-rheumatic drugs; CRP: C-reactive protein; FFMI: fat-free mass index; BASDAI: Bath Ankylosing Spondylitis Disease Activity Index; ASDAS-CRP: Ankylosing Spondylitis Disease Activity Score with C-reactive Protein; LDA: low disease activity; HDA: high disease activity; VHDA: very high disease activity; BASFI: Bath Ankylosing Spondylitis Functional Index; ASAS-HI: Assessment of Spondyloarthritis International Society Health Index; SF-12: Short Form Health Survey.

**Table 2:** Differences between patients without and with presarcopenia.

	Without Presarcopenia (*n* = 72)	With Presarcopenia (*n* = 22)	*p*
Sociodemographic and anthropometric data			
Male	55 (76.4%)	14 (63.6%)	ns
Mean age (years)	63.8 ± 8.4	66.1 ± 11.1	ns
BMI (kg/m^2^)	29.1 ± 4.2	24.7 ± 4.1	<0.01
Normal	5 (6.9%)	11 (50%)	ns
Overweight	39 (54.2%)	9 (40.9%)	ns
Obese	28 (38.9%)	2 (9.1%)	ns
Never smokers	37 (51.4%)	13 (59.1%)	ns
Regular exercise	19 (26.4%)	3 (13.6%)	ns
Fragility fracture	5 (6.9%)	1 (4.5%)	ns
axSpA and sarcopenia assessment			
Disease duration (months)	24.9 ± 13.9	29.8 ± 12.7	ns
r-axSpA	53 (73.6%)	21 (95.5%)	ns
HLA-B27-positive	56 (77.8%)	18 (81.8%)	ns
Articular symptoms			ns
Peripheral arthritis	31 (43.1%)	11 (50%)	
Enthesitis	20 (27.8%)	4 (18.2%)	
Dactylitis	3 (4.2%)	2 (9.1%)	
Extra-articular symptoms			ns
Uveitis	19 (26.4%)	9 (40.9%)	
Psoriasis	10 (13.9%)	1 (4.5%)	
Onychopathy	2 (2.8%)	0	
IBD	11 (15.3%)	1 (4.5%)	
Current treatment			
NSAID	49 (68.1%)	17 (77.3%)	ns
Glucocorticoids	5 (6.9%)	1 (4.5%)	ns
csDMARDs	4 (5.6%)	0	ns
bDMARDs	44 (61.1%)	7 (31.8%)	0.03
Hemoglobin (g/L)	146.8 ± 17.4	143.3 ± 13.8	ns
CRP (mg/L)	3.8 ± 7.9	2.4 ± 2.5	ns
FFMI	19.6 ± 2.6	16.1 ± 2	<0.01
BASDAI	3.6 ± 2.2	3.3 ± 2.2	ns
ASDAS-CRP	2.1 ± 0.9	1.2 ± 0.9	ns
Inactive	13 (18.1%)	5 (22.7%)	
LDA	27 (37.5%)	7 (31.8%)	
HDA	26 (36.1%)	8 (36.4%)	
VHDA	6 (8.3%)	2 (9.1%)	
BASFI	3.8 ± 2.5	3.7 ± 2.8	ns
ASAS-HI	5.8 ± 3.8	5.7 ± 3.9	ns
SF-12			ns
Mental health	50 ± 11.2	48.9 ± 11.7	
Physical health	40.3 ± 10.1	40.7 ± 12.9	
SARC-F	2.4 ± 2.2	2 ± 2	ns

Results are expressed as *n* (%) or mean ± SD. BMI: body mass index; axSpA: axial spondyloarthritis; r-axSpA: radiographic axial spondyloarthritis; IBD: inflammatory bowel disease; NSAID: nonsteroidal anti-inflammatory drugs; csDMARDs: conventional synthetic disease-modifying anti-rheumatic drugs; bDMARDs: biologic disease-modifying anti-rheumatic drugs; CRP: C-reactive protein; FFMI: fat-free mass index; BASDAI: Bath Ankylosing Spondylitis Disease Activity Index; ASDAS-CRP: Ankylosing Spondylitis Disease Activity Score with C-reactive Protein; LDA: low disease activity; HDA: high disease activity; VHDA: very high disease activity; BASFI: Bath Ankylosing Spondylitis Functional Index; ASAS-HI: Assessment of Spondyloarthritis International Society Health Index; SF-12: Short Form Health Survey.

**Table 3:** Sensitivity and specificity of SARC-F with predictive values and diagnostic accuracy.

	Sensitivity	Specificity	PV+	PV−	Diagnostic Accuracy
Sarcopenia	100.0	75.8	12	100	76.6

PV+: positive predictive value; PV−: negative predictive value.

## Data Availability

The data presented in this study are available on request from the corresponding author due to ethical reasons.

## Abbreviations

The following abbreviations are used in this manuscript:

axSpA	axial spondyloarthritis
AS	ankylosing spondylitis
EWGSOP	European Working Group on Sarcopenia in Older People
ASAS	Assessment of Spondyloarthritis International Society
DXA	whole-body densitometry
BMI	body mass index
FFMI	fat-free mass index
NSAIDs	nonsteroidal anti-inflammatory drugs
csDMARDs	conventional synthetic disease-modifying anti-rheumatic drugs
bDMARDs	biologic disease-modifying anti-rheumatic drugs
CRP	C-reactive protein
BASDAI	Bath Ankylosing Spondylitis Disease Activity Index
ASDAS-CRP	Ankylosing Spondylitis Disease Activity Score with C-reactive Protein
BASFI	Bath Ankylosing Spondylitis Functional Index
ASAS-HI	ASAS Health Index
SF-12	Short-Form Health Survey
SMI	skeletal mass index
PPV	positive predictive value
NPV	negative predictive value
r-axSpA	radiographic axial spondyloarthritis
IBD	inflammatory bowel disease
LDA	low disease activity
HDA	high disease activity
VHDA	very high disease activity
